# Supplementary material for: Heparin-based hydrogel scaffolding alters the transcriptomic profile and increases the chemoresistance of MDA-MB-231 triple-negative breast cancer cells
Source: Biomater Sci. 2020 Feb 13;8(10):2786–96. doi: 10.1039/c9bm01481k (PMC7497406; doi:10.1039/c9bm01481k)
Supplement: Supplementary file 2 [file BM-008-C9BM01481K-s002.zip › Supplementary File 4/HP-B hydrogel + EGF (s)vControl/Pathways/my_analysis.Gsea.1545201170987/HALLMARK_APICAL_JUNCTION.html]

Details for gene set HALLMARK\_APICAL\_JUNCTION[GSEA]

|  || Dataset | expr.class.cls#HYDEGF\_versus\_CONTROL.class.cls#HYDEGF\_versus\_CONTROL\_repos |
| Phenotype | class.cls#HYDEGF\_versus\_CONTROL\_repos |
| Upregulated in class | CONTROL |
| GeneSet | HALLMARK\_APICAL\_JUNCTION |
| Enrichment Score (ES) | -0.22911033 |
| Normalized Enrichment Score (NES) | -1.0519112 |
| Nominal p-value | 0.33333334 |
| FDR q-value | 0.3924778 |
| FWER p-Value | 1.0 |
Table: GSEA Results Summary

  

Fig 1: Enrichment plot: HALLMARK\_APICAL\_JUNCTION      
 Profile of the Running ES Score & Positions of GeneSet Members on the Rank Ordered List

  

| PROBE | DESCRIPTION (from dataset) | GENE SYMBOL | GENE\_TITLE | RANK IN GENE LIST | RANK METRIC SCORE | RUNNING ES | CORE ENRICHMENT || 1 | NEGR1 | na |  |  | 527 | 2.094 | -0.0100 | No |
| 2 | CDH4 | na |  |  | 680 | 1.921 | -0.0018 | No |
| 3 | ATP1A3 | na |  |  | 866 | 1.773 | 0.0034 | No |
| 4 | CLDN15 | na |  |  | 988 | 1.697 | 0.0113 | No |
| 5 | ACTN1 | na |  |  | 1089 | 1.628 | 0.0198 | No |
| 6 | NF2 | na |  |  | 1227 | 1.555 | 0.0257 | No |
| 7 | AMIGO2 | na |  |  | 1348 | 1.495 | 0.0319 | No |
| 8 | BAIAP2 | na |  |  | 1394 | 1.470 | 0.0420 | No |
| 9 | EGFR | na |  |  | 1395 | 1.470 | 0.0543 | No |
| 10 | VAV2 | na |  |  | 1446 | 1.450 | 0.0639 | No |
| 11 | COL17A1 | na |  |  | 1604 | 1.390 | 0.0674 | No |
| 12 | PARD6G | na |  |  | 1907 | 1.291 | 0.0624 | No |
| 13 | AMIGO1 | na |  |  | 1996 | 1.267 | 0.0684 | No |
| 14 | HRAS | na |  |  | 2033 | 1.257 | 0.0771 | No |
| 15 | MYH10 | na |  |  | 2056 | 1.250 | 0.0865 | No |
| 16 | COL16A1 | na |  |  | 2059 | 1.249 | 0.0969 | No |
| 17 | EVL | na |  |  | 2340 | 1.167 | 0.0920 | No |
| 18 | PLCG1 | na |  |  | 2414 | 1.148 | 0.0979 | No |
| 19 | MAP4K2 | na |  |  | 2423 | 1.146 | 0.1071 | No |
| 20 | PTK2 | na |  |  | 2705 | 1.079 | 0.1014 | No |
| 21 | SYMPK | na |  |  | 2723 | 1.074 | 0.1096 | No |
| 22 | AKT2 | na |  |  | 2780 | 1.056 | 0.1155 | No |
| 23 | TSPAN4 | na |  |  | 2851 | 1.040 | 0.1206 | No |
| 24 | WNK4 | na |  |  | 2855 | 1.039 | 0.1292 | No |
| 25 | MAPK11 | na |  |  | 3052 | 0.992 | 0.1273 | No |
| 26 | AMH | na |  |  | 3116 | 0.977 | 0.1322 | No |
| 27 | PKD1 | na |  |  | 3133 | 0.973 | 0.1395 | No |
| 28 | IKBKG | na |  |  | 3228 | 0.954 | 0.1426 | No |
| 29 | LDLRAP1 | na |  |  | 3299 | 0.943 | 0.1469 | No |
| 30 | ZYX | na |  |  | 3364 | 0.929 | 0.1514 | No |
| 31 | TAOK2 | na |  |  | 3626 | 0.880 | 0.1451 | No |
| 32 | NLGN3 | na |  |  | 3739 | 0.857 | 0.1464 | No |
| 33 | SHROOM2 | na |  |  | 3748 | 0.856 | 0.1532 | No |
| 34 | CLDN14 | na |  |  | 3851 | 0.838 | 0.1549 | No |
| 35 | MADCAM1 | na |  |  | 3996 | 0.812 | 0.1542 | No |
| 36 | SPEG | na |  |  | 4116 | 0.794 | 0.1546 | No |
| 37 | RASA1 | na |  |  | 4413 | 0.747 | 0.1454 | No |
| 38 | IRS1 | na |  |  | 4622 | 0.716 | 0.1405 | No |
| 39 | CLDN9 | na |  |  | 4687 | 0.709 | 0.1431 | No |
| 40 | DLG1 | na |  |  | 4883 | 0.670 | 0.1385 | No |
| 41 | VASP | na |  |  | 5062 | 0.643 | 0.1346 | No |
| 42 | NF1 | na |  |  | 5105 | 0.636 | 0.1377 | No |
| 43 | PIK3R3 | na |  |  | 5161 | 0.627 | 0.1401 | No |
| 44 | TSC1 | na |  |  | 5185 | 0.623 | 0.1441 | No |
| 45 | MAPK14 | na |  |  | 5197 | 0.620 | 0.1488 | No |
| 46 | INPPL1 | na |  |  | 5238 | 0.615 | 0.1519 | No |
| 47 | EXOC4 | na |  |  | 5249 | 0.613 | 0.1565 | No |
| 48 | JAM3 | na |  |  | 5275 | 0.610 | 0.1603 | No |
| 49 | EPB41L2 | na |  |  | 5330 | 0.599 | 0.1625 | No |
| 50 | NLGN2 | na |  |  | 5487 | 0.577 | 0.1592 | No |
| 51 | ICAM2 | na |  |  | 5502 | 0.576 | 0.1633 | No |
| 52 | PCDH1 | na |  |  | 5546 | 0.571 | 0.1659 | No |
| 53 | CD274 | na |  |  | 5686 | 0.548 | 0.1632 | No |
| 54 | GAMT | na |  |  | 5843 | 0.525 | 0.1594 | No |
| 55 | VCL | na |  |  | 6326 | 0.460 | 0.1380 | No |
| 56 | STX4 | na |  |  | 6377 | 0.454 | 0.1392 | No |
| 57 | FBN1 | na |  |  | 6535 | 0.433 | 0.1346 | No |
| 58 | LAYN | na |  |  | 6621 | 0.421 | 0.1337 | No |
| 59 | CLDN18 | na |  |  | 6759 | 0.401 | 0.1299 | No |
| 60 | LIMA1 | na |  |  | 6837 | 0.392 | 0.1291 | No |
| 61 | MVD | na |  |  | 6948 | 0.379 | 0.1265 | No |
| 62 | NEXN | na |  |  | 7344 | 0.323 | 0.1085 | No |
| 63 | VCAN | na |  |  | 7533 | 0.301 | 0.1012 | No |
| 64 | PARVA | na |  |  | 7660 | 0.281 | 0.0969 | No |
| 65 | ICAM5 | na |  |  | 7918 | 0.253 | 0.0856 | No |
| 66 | GNAI1 | na |  |  | 7972 | 0.246 | 0.0849 | No |
| 67 | CDSN | na |  |  | 8156 | 0.230 | 0.0772 | No |
| 68 | DHX16 | na |  |  | 8203 | 0.223 | 0.0766 | No |
| 69 | TJP1 | na |  |  | 8311 | 0.208 | 0.0728 | No |
| 70 | MYL9 | na |  |  | 8322 | 0.207 | 0.0740 | No |
| 71 | TUBG1 | na |  |  | 8505 | 0.186 | 0.0660 | No |
| 72 | PPP2R2C | na |  |  | 8749 | 0.158 | 0.0546 | No |
| 73 | MAPK13 | na |  |  | 8783 | 0.153 | 0.0541 | No |
| 74 | SHC1 | na |  |  | 8800 | 0.150 | 0.0546 | No |
| 75 | THBS3 | na |  |  | 8805 | 0.148 | 0.0556 | No |
| 76 | TNFRSF11B | na |  |  | 8824 | 0.144 | 0.0559 | No |
| 77 | BMP1 | na |  |  | 9326 | 0.085 | 0.0303 | No |
| 78 | SIRPA | na |  |  | 9518 | 0.072 | 0.0209 | No |
| 79 | CLDN11 | na |  |  | 9685 | 0.051 | 0.0126 | No |
| 80 | LAMB3 | na |  |  | 10015 | 0.019 | -0.0045 | No |
| 81 | TIAL1 | na |  |  | 10113 | 0.003 | -0.0096 | No |
| 82 | AKT3 | na |  |  | 10132 | 0.001 | -0.0105 | No |
| 83 | CDK8 | na |  |  | 10247 | -0.016 | -0.0164 | No |
| 84 | MPP5 | na |  |  | 10543 | -0.042 | -0.0315 | No |
| 85 | CRAT | na |  |  | 10668 | -0.062 | -0.0375 | No |
| 86 | SRC | na |  |  | 10830 | -0.082 | -0.0452 | No |
| 87 | GRB7 | na |  |  | 10907 | -0.096 | -0.0484 | No |
| 88 | ITGB4 | na |  |  | 11077 | -0.118 | -0.0563 | No |
| 89 | ADAM23 | na |  |  | 11119 | -0.118 | -0.0575 | No |
| 90 | ADAM15 | na |  |  | 11195 | -0.122 | -0.0604 | No |
| 91 | PBX2 | na |  |  | 11295 | -0.136 | -0.0644 | No |
| 92 | YWHAH | na |  |  | 11375 | -0.143 | -0.0674 | No |
| 93 | CD276 | na |  |  | 11554 | -0.169 | -0.0753 | No |
| 94 | HADH | na |  |  | 11730 | -0.192 | -0.0829 | No |
| 95 | ADRA1B | na |  |  | 11742 | -0.194 | -0.0818 | No |
| 96 | CNN2 | na |  |  | 11756 | -0.197 | -0.0808 | No |
| 97 | SDC3 | na |  |  | 11878 | -0.214 | -0.0854 | No |
| 98 | CDH3 | na |  |  | 11901 | -0.214 | -0.0847 | No |
| 99 | GTF2F1 | na |  |  | 12553 | -0.290 | -0.1165 | No |
| 100 | CDH15 | na |  |  | 13079 | -0.352 | -0.1410 | No |
| 101 | ADAM9 | na |  |  | 13107 | -0.354 | -0.1395 | No |
| 102 | CD34 | na |  |  | 13109 | -0.355 | -0.1365 | No |
| 103 | VCAM1 | na |  |  | 13271 | -0.382 | -0.1418 | No |
| 104 | SORBS3 | na |  |  | 13406 | -0.404 | -0.1454 | No |
| 105 | LAMA3 | na |  |  | 13513 | -0.415 | -0.1475 | No |
| 106 | WASL | na |  |  | 13566 | -0.423 | -0.1467 | No |
| 107 | CDH1 | na |  |  | 13575 | -0.423 | -0.1435 | No |
| 108 | PFN1 | na |  |  | 13723 | -0.445 | -0.1475 | No |
| 109 | TMEM8B | na |  |  | 14230 | -0.509 | -0.1698 | No |
| 110 | CERCAM | na |  |  | 14360 | -0.531 | -0.1721 | No |
| 111 | CTNNA1 | na |  |  | 14530 | -0.560 | -0.1762 | No |
| 112 | NRTN | na |  |  | 14832 | -0.609 | -0.1869 | No |
| 113 | FSCN1 | na |  |  | 14874 | -0.615 | -0.1839 | No |
| 114 | JUP | na |  |  | 15173 | -0.664 | -0.1939 | No |
| 115 | RHOF | na |  |  | 15197 | -0.669 | -0.1895 | No |
| 116 | CX3CL1 | na |  |  | 15637 | -0.749 | -0.2062 | No |
| 117 | ARPC2 | na |  |  | 15866 | -0.794 | -0.2115 | No |
| 118 | PIK3CB | na |  |  | 15970 | -0.815 | -0.2101 | No |
| 119 | ITGA10 | na |  |  | 16334 | -0.907 | -0.2215 | Yes |
| 120 | RRAS | na |  |  | 16340 | -0.908 | -0.2141 | Yes |
| 121 | PTEN | na |  |  | 16477 | -0.941 | -0.2133 | Yes |
| 122 | CD99 | na |  |  | 16501 | -0.948 | -0.2065 | Yes |
| 123 | MPZL1 | na |  |  | 16703 | -1.000 | -0.2087 | Yes |
| 124 | TGFBI | na |  |  | 16713 | -1.002 | -0.2007 | Yes |
| 125 | TRAF1 | na |  |  | 16954 | -1.073 | -0.2043 | Yes |
| 126 | MPZL2 | na |  |  | 17051 | -1.103 | -0.2000 | Yes |
| 127 | ICAM1 | na |  |  | 17147 | -1.127 | -0.1955 | Yes |
| 128 | RAC2 | na |  |  | 17178 | -1.137 | -0.1875 | Yes |
| 129 | CTNND1 | na |  |  | 17207 | -1.147 | -0.1794 | Yes |
| 130 | INSIG1 | na |  |  | 17226 | -1.153 | -0.1706 | Yes |
| 131 | GNAI2 | na |  |  | 17280 | -1.174 | -0.1635 | Yes |
| 132 | SLIT2 | na |  |  | 17519 | -1.266 | -0.1653 | Yes |
| 133 | RSU1 | na |  |  | 17803 | -1.387 | -0.1685 | Yes |
| 134 | CLDN4 | na |  |  | 17975 | -1.476 | -0.1651 | Yes |
| 135 | B4GALT1 | na |  |  | 18015 | -1.493 | -0.1545 | Yes |
| 136 | CLDN7 | na |  |  | 18127 | -1.564 | -0.1472 | Yes |
| 137 | MYL12B | na |  |  | 18144 | -1.575 | -0.1348 | Yes |
| 138 | CALB2 | na |  |  | 18210 | -1.607 | -0.1247 | Yes |
| 139 | CAP1 | na |  |  | 18502 | -1.837 | -0.1245 | Yes |
| 140 | SKAP2 | na |  |  | 18509 | -1.844 | -0.1093 | Yes |
| 141 | LAMC2 | na |  |  | 18772 | -2.148 | -0.1049 | Yes |
| 142 | ARHGEF6 | na |  |  | 18833 | -2.255 | -0.0891 | Yes |
| 143 | CDH11 | na |  |  | 18932 | -2.418 | -0.0739 | Yes |
| 144 | ITGA2 | na |  |  | 18964 | -2.492 | -0.0545 | Yes |
| 145 | SGCE | na |  |  | 18996 | -2.570 | -0.0345 | Yes |
| 146 | PECAM1 | na |  |  | 19026 | -2.661 | -0.0137 | Yes |
| 147 | MDK | na |  |  | 19037 | -2.689 | 0.0085 | Yes |
Table: GSEA details [plain text format]

  

Fig 2: HALLMARK\_APICAL\_JUNCTION      
 Blue-Pink O' Gram in the Space of the Analyzed GeneSet

  

Fig 3: HALLMARK\_APICAL\_JUNCTION: Random ES distribution      
 Gene set null distribution of ES for **HALLMARK\_APICAL\_JUNCTION**
